# Fiberweb: Diffusion Visualization and Processing in the Browser

**DOI:** 10.3389/fninf.2017.00054

**Published:** 2017-08-18

**Authors:** Louis-Philippe Ledoux, Felix C. Morency, Martin Cousineau, Jean-Christophe Houde, Kevin Whittingstall, Maxime Descoteaux

**Affiliations:** ^1^Sherbrooke Connectivity Imaging Lab, Computer Science Department, Faculty of Science, University of Sherbrooke Sherbrooke, QC, Canada; ^2^Imeka Sherbrooke, QC, Canada; ^3^Videos & Images Theory and Analytics Laboratory, University of Sherbrooke Sherbrooke, QC, Canada; ^4^Centre de Recherche CHUS, University of Sherbrooke Sherbrooke, QC, Canada; ^5^Department of Nuclear Medecine and Radiobiology, University of Sherbrooke Sherbrooke, QC, Canada; ^6^Department of Diagnostic Radiology, University of Sherbrooke Sherbrooke, QC, Canada

**Keywords:** diffusion MRI, tractography, medical visualization, interaction, web, WebGL

## Abstract

Data visualization is one of the most important tool to explore the brain as we know it. In this work, we introduce a novel browser-based solution for medical imaging data visualization and interaction with diffusion-weighted magnetic resonance imaging (dMRI) and tractography data: Fiberweb. It uses a recent technology, WebGL, that has yet to be fully explored for medical imaging purposes. There are currently very few software tools that allow medical imaging data visualization in the browser, and none of these tools support efficient data interaction and processing, such as streamlines selection and real-time deterministic and probabilistic tractography (RTT). With Fiberweb allowing these types of interaction, it is no longer the case. We show results of the visualization of medical imaging data, and demonstrate that our new RTT probabilistic algorithm can compare to a state of the art offline algorithm. Overall, Fiberweb pushes the boundary of interaction combined with scientific visualization, which opens great perspectives for quality control and neurosurgical navigation on browser-based mobile and static devices.

## 1. Introduction

In recent years, the world has seen a huge increase in the use of mobile devices and web technologies. It is becoming more and more important that all resources are always available at anytime, anywhere (e.g., Dropbox, GoogleDrive, iCloud, OneDrive, and many other such providers). Currently, in the field of medical imaging, there are a lot of software tools to view and interact with data. However, only a few exist for the browser, even with all the recent possibilities that the Web Graphics Library (WebGL) enables. Having the possibility to simply load a web page to start interacting with brain imaging data would open possibilities for the whole field.

There are a few existing software tools that are currently available and offer visualization of medical imaging data in the web browser. First, there is Slice:Drop (Haehn, 2013). It allows its users to visualize anatomies, streamlines and volumes that are on their computer, and the file types it supports cover most of the standard medical imaging formats (e.g., NIFTI, TrackVis, .trk, etc.). Then, there are two web applications that were developed by the Max Planck Institute. In 2011, they made an interactive web page covering an article by Friederici (Friederici, 2011; Friederici et al., 2012). It allows the user to view the various fiber bundles presented in said article. In 2014, they developed Brain Networks (Heuer et al., 2014), which allows exploring structural and functional connectivity data provided by the application. Finally, the AFQ Browser (Richie-Halford et al., 2016) was created to visualize the results obtained by Automated Fiber Quantification (AFQ; Yeatman et al., 2012). Specific results can be selected from desired white matter bundles and subjects. All of those tools are strictly for visualization. An interesting evolution would be to allow interaction with the data. For instance, one could want to virtually dissect a tractogram, or rapidly explore tractography results of a tumor patient in real-time.

In this work, we propose *Fiberweb*, a web-based visualization tool with additional processing and interaction capabilities. The contributions of this work are:

- Full-brain tractogram (up to 75,000 “streamlines”) loading and smooth interaction using a selection object. This enables virtual dissection manipulations directly in Fiberweb.
- Real-time web-based fiber tractography.
- A novel probabilistic algorithm for real-time tracking, which is the main contribution.

These features allow the user to interact and process his data in ways that were not previously possible in the browser. This means no installation is required for the user, and no server is required for backend computation. This opens great perspectives for quality control and neurosurgical navigation on browser-based mobile and static devices.

## 2. Methods

### 2.1. Visualization in the web

WebGL is the driving technology that enabled the creation of Fiberweb. WebGL's creation was made possible by the arrival of HTML5, and more specifically one of its new features, the canvas element. This element allows the creation of a drawable region in the browser, which can then be modified using JavaScript. WebGL is a low-level graphics library for 3D rendering in the browser and is functional with all the major players, such as Chrome, Firefox, Edge and Safari.

#### 2.1.1. Three.js

To simplify the development of Fiberweb, a high-level library called Three.js (Cabello, 2010) was used. This library wraps WebGL calls and makes it more convenient to use shaders, manage scenes and render primitives (e.g., lines). According to stats.js.org (as of january 2017), Three.js is one of the most used WebGL library (it is used by AFQ Browser), and has a very active community, leading to constant updates. This will make it easier to upgrade Fiberweb over time. It is also lower level than the X toolkit (Haehn et al., 2014, used by Slice:drop), making it a better choice for this implementation, since basic objects need to be directly manipulable for performance reasons.

#### 2.1.2. Visualization techniques

**Figure 1:**
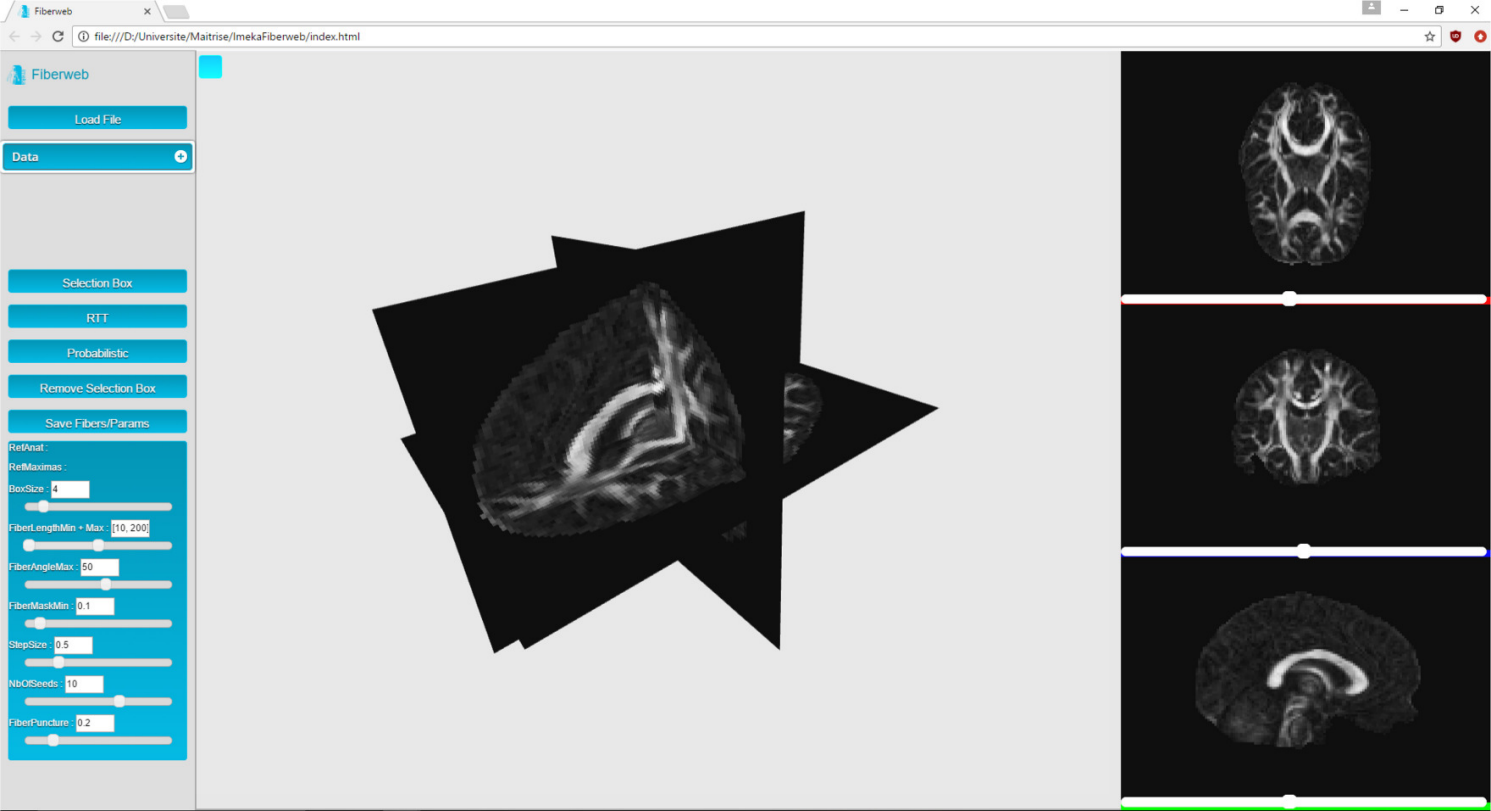
Anatomy visualization in 3D and 2D views inside Fiberweb.

Fiberweb can currently display three types of medical imaging data: anatomies (peaks) and streamlines. These datasets can be visualized both from a 3D perspective as well as a 2D one, as seen in Figure 1. This is implemented using four different renderers, one for the three dimensional view, and three for two dimensional views, representing the axial, coronal and sagittal views (Figure 1). The 3D view can be manipulated through translations, rotations and zooms. For the 2D views, only translations and zooms are available (see video at www.imeka.ca/fiberweb).

“Anatomies” are any 3D volume datasets, such as T1, T2, fractional anisotropy (FA), mean diffusivity (MD), and colored FA (4D image). They are represented by plane buffer geometries. These are simple textured planes, both in 3D and 2D. These textures are computed during the initialization. They can be navigated through with the help of different sliders for each view. When a slice change is made, planes are simply translated, and a simple texture swap is applied since they are already loaded in memory.

**Figure 2:**
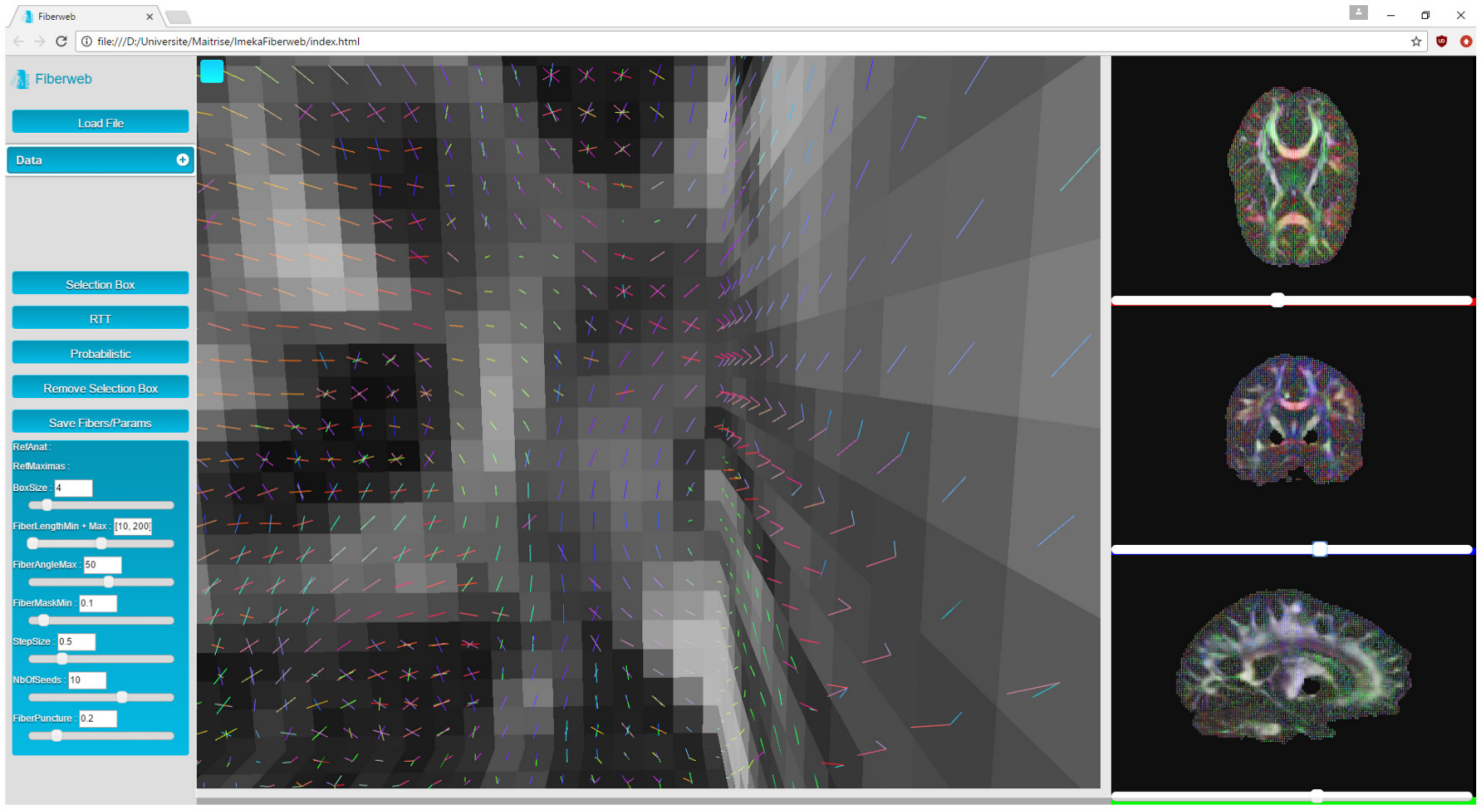
Anatomy and peaks visualization in 3D and 2D views inside Fiberweb.

Next are the “peaks,” which are 4D volumes. The first three represent the coordinates of a peak (X, Y, Z). The fourth one contains the directions extracted from the fiber ODFs (X, Y, Z, N where *N* = (*x*_1_, *y*_1_, *z*_1_, …, *x*_5_, *y*_5_, *z*_5_)). Up to 5 directions can be kept per fiber ODF. The peaks can have an additional α parameter to represent the maxima's uncertainty (described later for probabilistic tracking, see Figure **5**) if the dataset is probabilistic (X, Y, Z, α). Peaks are represented by another object provided by Three.js called line segments (Figure 2). However, the geometry as well as the colors need to be recomputed for each new rendered slice, since this is computed only for the currently displayed slice (see video at www.imeka.ca/fiberweb).

**Figure 3:**
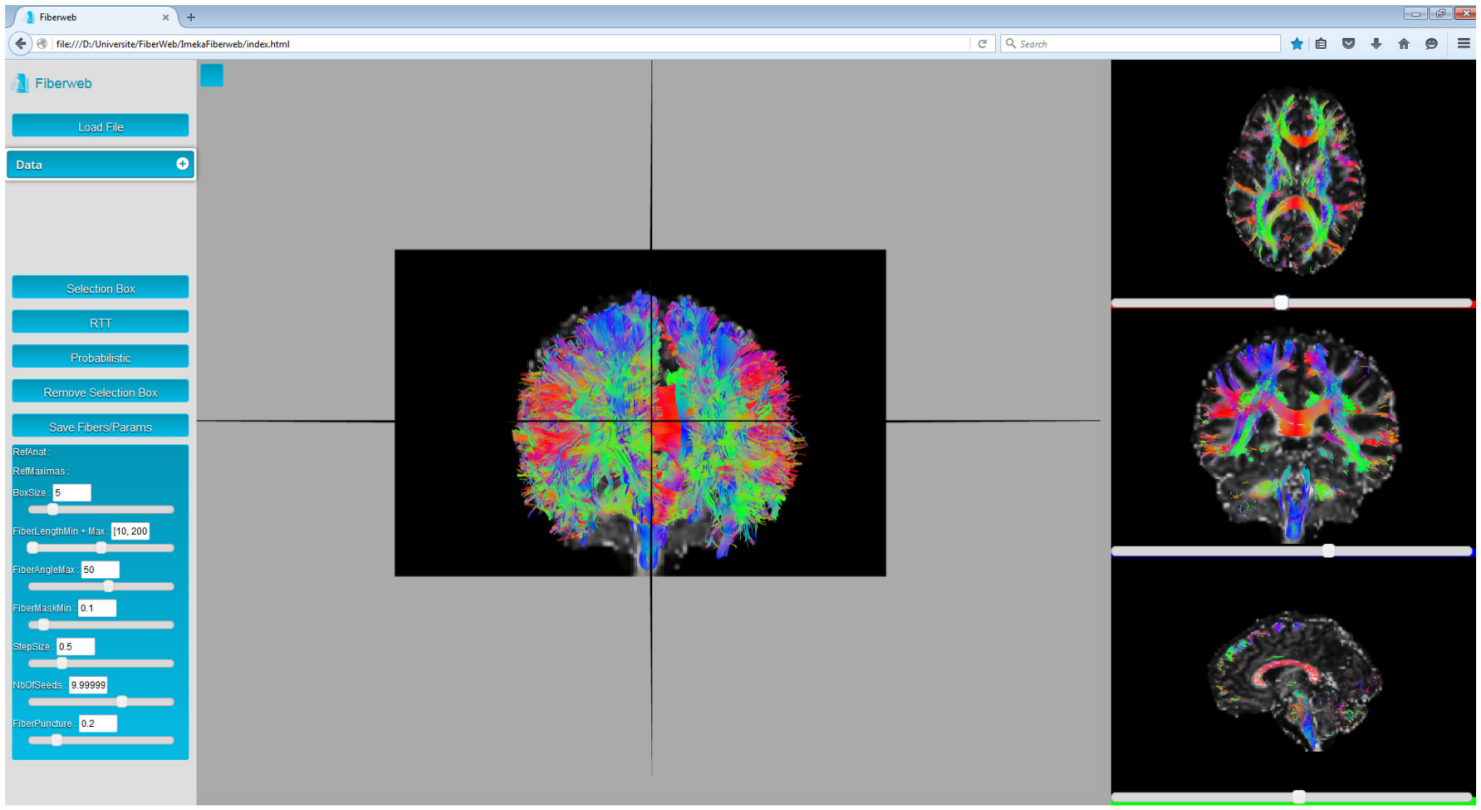
Anatomy and streamlines visualization inside Fiberweb, with the intersected streamlines in the 2D views.

Finally, there are the “streamlines”. A streamline is a set of 3D points (*p*_*i*_ = (*x*_*p*_*i*__, *y*_*p*_*i*__, *z*_*p*_*i*__), where *i* ∈ (1, *N*) and *N* is the number of points in the streamline). The distance between two consecutive points is called the step size, and is usually regular, though not required. Each pair of points of a streamline is represented by a line segment. During the initialization process, the color for each segment is computed. In the 3D view, line segments are simply drawn. However, in the multiple 2D views available, only the part of the streamlines matching the currently displayed anatomy slice are drawn. This is implemented through a shader which calculates the volume in which lines will be displayed, as seen on Figure 3. This also means that whenever the slice is changed, only the parameters sent to the shader are changed, making it extremely efficient and fast. As it will be discussed later, Fiberweb currently works best with streamlines datasets of up to 75,000 streamlines, which on average have 100 points per streamline. To sum up, there are currently two ways to visualize streamlines: viewing all the streamlines in 3D, or viewing only the intersected streamlines with the planes of the anatomies in the 2D views, as seen in Figure 3 (see video at www.imeka.ca/fiberweb).

### 2.2. Interactions and processing

Fiberweb supports various interaction mechanisms: streamlines selection and real-time tractography.

#### 2.2.1. Selection

Fiberweb provides a streamlines selection mechanism based on a points inclusion test. All streamlines having at least one point inside the selection box will be displayed.

To reduce complexity and increase performance (trying to ensure that feedback is provided in real time), an octree is used to classify streamlines points. An octree is a data structure that recursively subdivides space in eight smaller regions until a certain level of simplification is obtained. Once the space is divided, each point is classified into the octree. This results in a much smaller search space and fewer points to search through.

Even though an octree was already available in Three.js, a new implementation was done in Fiberweb. The main reason was that the octree of Three.js dynamically transforms the octree every time a new element is added to it. Since a full dataset of streamlines contains a high number of points, it was unnecessarily slow to initialize, especially since the position of the points is static and known a priori.

#### 2.2.2. Menu and parameters

**Figure 4:**
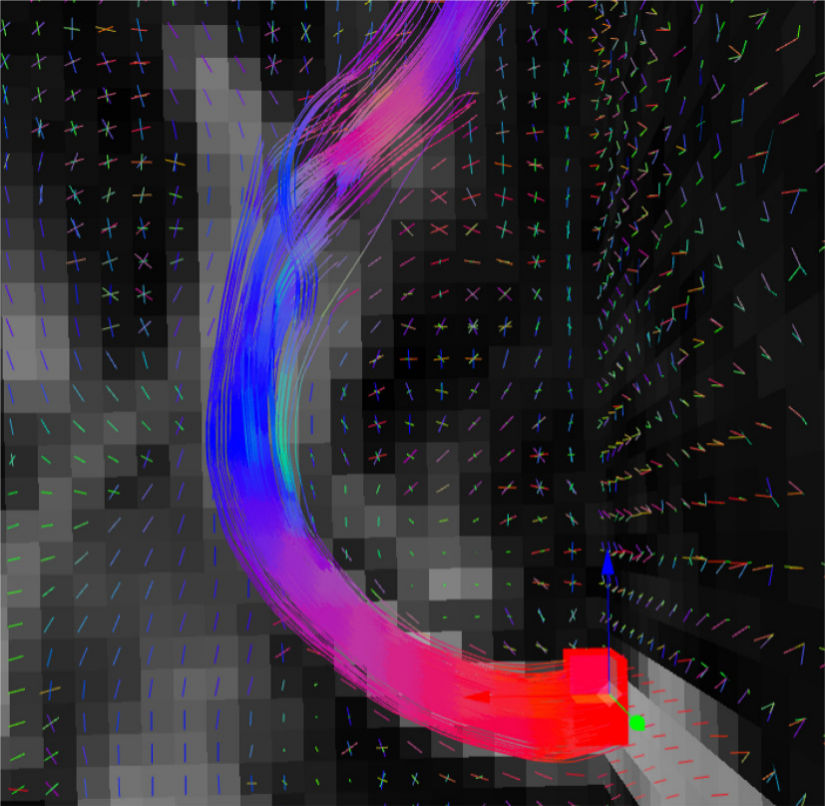
Fiber bundle (part of the corpus callosum), result of the real-time tractography, with the initialization box (red) in the mid-sagittal slice of the CC and the following parameters: angle threshold of 50°, fiber length minimum of 10 mm and maximum of 200 mm, FA threshold of 0.1, a step size of 0.5 mm, 10 seeds per axis and a puncture value (g) of 0.2.

From the data drop-down menu (see Figure 1), the user can select which anatomy and which map of peaks are selected as a reference for the real-time tractography. The box size can be modified by the user both for the selection and the tractography, as seen on Figure 4. The rest of the parameters are specifically for the latter (Chamberland et al., 2014). Fiber length changes the minimum and maximum span a fiber must have to be valid. The fiber angle is the maximum angle a new direction can have with the previous one. The fiber mask parameter is the minimum value the voxel needs to have to continue tracking. The step size is the distance the tracking travels at every step of the algorithm. The number of seeds determines the quantity of streamlines the results will have. Finally, the fiber puncture is a variable that weighs the incoming direction of the tracking as well as the next one (Chamberland et al., 2014). The equation that the deterministic RTT algorithm follows is:

(1) $$V_{n+1}=(fV_{n})+(1-f)((1-g)V_{n-1}+gV_{n}),$$

where f is the FA (or GFA, AFD, or any other underlying scalar map or mask) at the current voxel, g is the puncture value that was mentionned previously and **V** ∈ ℝ^3^ is the direction (x, y, z) which the algorithm follows (*n* − 1 is the previous direction, *n* is the current direction and *n* + 1 is the next direction to follow).

Additionally, the user can save the computed/extracted streamlines, from both the selection and the real-time tractography. The parameters are also saved, making it easier for a user to recreate its selection or tracking.

#### 2.2.3. Deterministic real-time tractography (RTT)

Chamberland et al. (2014) deterministic RTT algorithm, which was originally implemented in the Fibernavigator (Vaillancourt et al., 2010), was implemented in Fiberweb. In order to use it, the first step is to load a precomputed map of peaks. These peaks are extracted from a map of fiber ODFs (Tournier et al., 2007; Descoteaux et al., 2009) or any other diffusion model (multi-compartment models, DSI, etc.) (Wedeen et al., 2008; Seunarine and Alexander, 2009; Descoteaux, 2015, etc.) and correspond to the maximas found in each voxel. Once the peaks are computed offline, the tracking starts by seeding randomly inside a small selection box. This seeding strategy is known as region-of-interest (ROI) seeding, which was chosen over complete seeding, which seeds everywhere in the tracking mask (Basser et al., 2000; Catani et al., 2002; Descoteaux, 2010). For each seed, a random peak is selected from the voxel at its current position. The tracking consists in taking the current position, comparing the current tracking direction with all the peaks of the current voxel, and selecting the one with the smallest angular difference. Then, the current direction as well as the next direction are weighted with a user-defined variable. The algorithm then moves a step in this resulting direction. These steps are repeated until various possible end criteria are met, such as fiber length. As can be seen at www.imeka.ca/fiberweb, one can change the tracking parameters in real time (FA threshold, step size, curvature θ angle, puncture). This allows one to explore his peaks in real time for quality assurance or exploration purposes. Chamberland et al. (2014) have shown the power of such an approach for surgical planning and exploring the white matter architecture around the tumor.

#### 2.2.4. Probabilistic RTT

**Figure 5:**
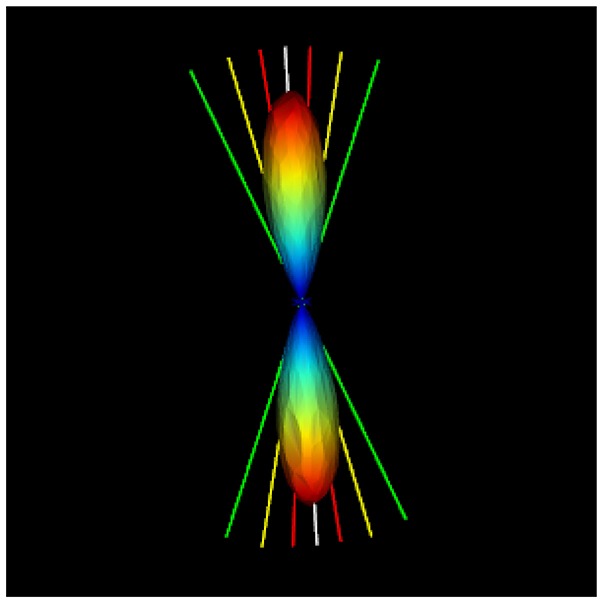
Illustration of the uncertainty alpha parameter at 95% (red), 75% (yellow), and 35% (green) of the maxima. The script to compute these alpha values for each fODF peak is given in the Appendix.

FiberWeb also introduces a new probabilistic real-time tracking technique, building on the already existing deterministic RTT technique. Instead of loading the standard peaks file containing only the directions of the maximas, the user needs to load a peaks file also containing the uncertainty α_*i*_ (X, Y, Z, N where *N* = (*x*_1_, *y*_1_, *z*_1_, α_1_, …, *x*_5_, *y*_5_, *z*_5_, α_5_)). The α_*i*_ could come from any source, but in this work, a Dipy script (Supplemental Material) that computes the full width half max (FWHM) of each fODF peak is provided. As seen in Figure 5, one can play with the percentage at which the FWHM is computed to have a more or less conservative α value around each fODF peak. To do this, the fODF is mapped onto a sphere of the user's choice, a 724 vertices symmetric one in our case (Garyfallidis et al., 2014). Then, starting from the different maximas of the sphere, the neighbouring vertices are traversed until reaching the desired fraction of the peak. The angle between that vertex and the one from the maxima is then computed and saved along with the peak.

The first part of the tracking algorithm is the same as for the deterministic version. Seeds are chosen randomly in the selection box. Then, a random peak associated to the voxel at the current position is chosen. Next, instead of simply following that tracking direction, the uncertainty angle associated with that peak is fetched. Then, a random direction in the “cone of uncertainty” formed by the uncertainty angle and the peak is chosen. Then, the algorithm continues executing normally, with the extra step of modifying the direction by an angle of uncertainty every time. Equation 1 then becomes:

(2) $$V_{n+1}=(f\cdot \theta (V_{n},\alpha ))+(1-f)((1-g)V_{n-1}+g\cdot \theta (V_{n},\alpha )),$$

where θ() represents the function modifying the direction **V** by picking a random direction in a cone formed by its uncertainty α. Pseudo-code of the implementation of the algorithm can be found in the annex.

#### 2.2.5. Dataset

The dataset used for the evaluation of the probabilistic tracking algorithm is found on the SCIL website (http://scil.dinf.usherbrooke.ca/?page_id=822&lang=en) and came originally from the Max Planck Institute (Anwander et al., 2007; Descoteaux et al., 2009). The data was collected on a 3T Magnetom Trio scanner (Siemens, Erlangen) using a spin-echo echo-planar-imaging sequence (TE/TR = 100 ms / 12 s) with a b-value of 1000 s/mm^2^ and 60 diffusion encoding gradients (Jones et al., 1999). The dimensions are 93 × 116 × 93 isotropic voxel resolution of 1.72 mm for both the T1-anatomy and the fODF map. The data comes from 8 healthy right-hand volunteers (4 males, 4 females), from whom written consent was obtained. fODFs were estimated with Dipy (Garyfallidis et al., 2014) using a maximal spherical harmonics order of 8. Peaks were extracted using a tessellated sphere with 724 points.

#### 2.2.6. Evaluation and validation

To determine the validity of our new real-time tracking algorithm, the evaluation was done in two ways, as similarly done in Chamberland et al. (2014). First, streamlines generated by our algorithm were qualitatively compared to the results of an offline probabilistic tracking algorithm, as implemented in Girard et al. (2014) and MRtrix (Tournier et al., 2012). The tracking was done on the same dataset. Then, a quantitative evaluation is done using the Dice coefficient (Dice, 1945), as done in Cousineau et al. (2016). Using the arcuate fasciculus, corpus callosum and corticospinal tract, the reconstructed bundles from our RTT algorithm vs the offline one are compared using a weighted version of the Dice coefficient to account for the density of streamlines traversing each voxel (see Supplemental Material for details). The evaluation of the framerate of the application was done on a laptop with the following system: Windows 8 64-bit, Video card (GPU): NVIDIA GeForce GTX 675MX (4GB), CPU: Intel(R) Core(TM) i7-3740QM @ 2.70 GHz (4 cores), 16 GB RAM.

## 3. Results

### 3.1. Visualization

In this work, it is considered that the baseline performance for the visualization part of the tool is to display an anatomical dataset. Displaying a peaks dataset does not affect the framerate in any noticeable way. In Figure 3, the Fiberweb interface is shown simultaneously displaying an anatomical dataset and a set of streamlines. In Figure 2, Fiberweb shows an anatomical dataset and its corresponding peaks dataset too. Currently, the 3D view directly allows for picking, interaction and selection. In the 2D renderers (axial/coronal/sagittal), streamlines intersecting with the anatomical dataset are shown (Figure 3).

**Figure 6:**
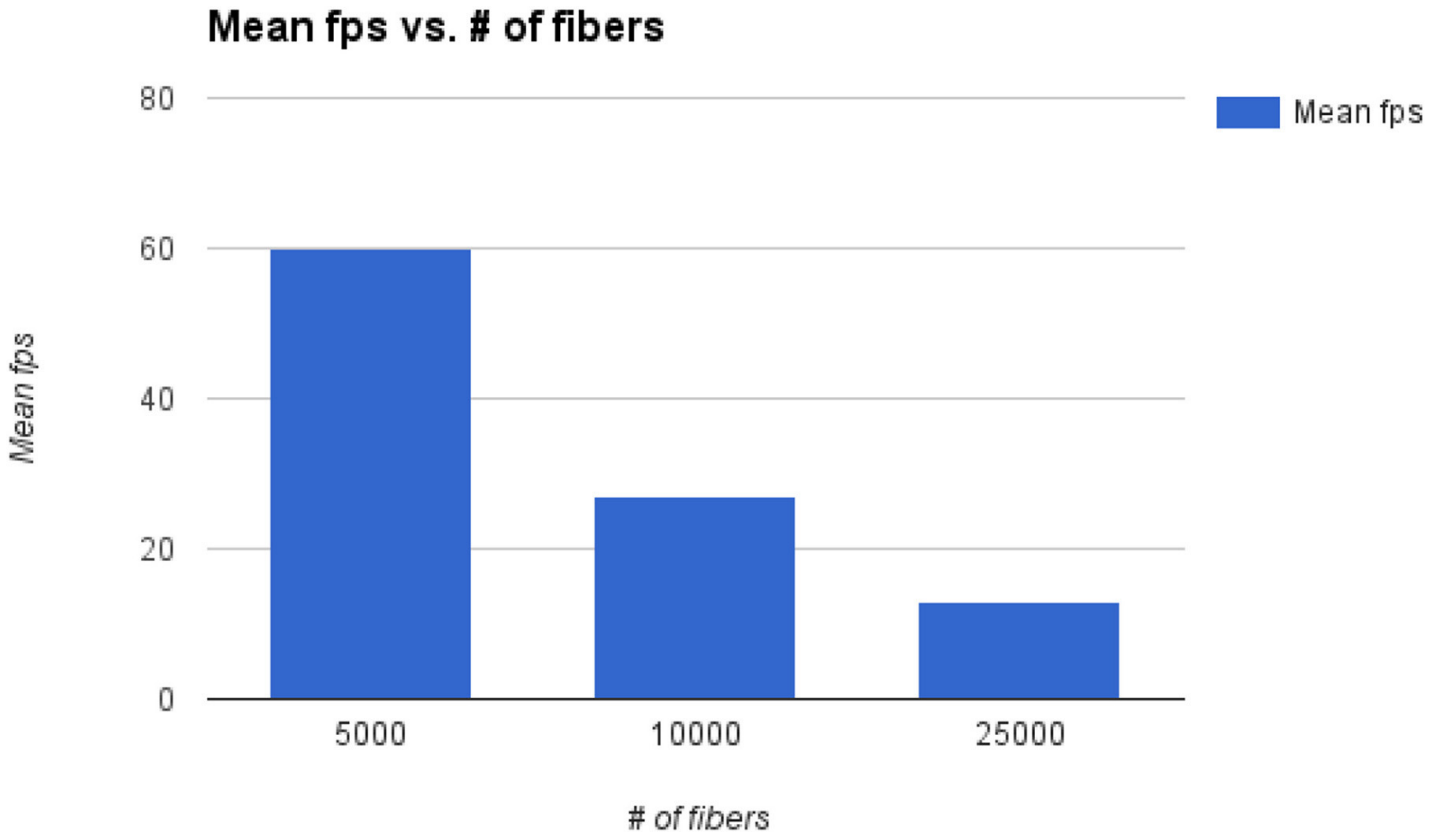
Mean framerate [in frames per second (fps)] vs. number of streamlines.

The main bottleneck of Fiberweb is the display of streamlines in general. The framerate and the maximal streamlines capacity are directly affected by the number of streamlines and the number of points per streamline. To avoid any potential bias, all the streamlines datasets that were used are raw and have roughly the same average number points per streamline (100 points). In Figure 6, it can be seen that doubling the number of streamlines approximately halves the framerate per second (fps). With 25,000 streamlines, the framerate stops being affected in a noticeable way, but memory issues become problematic. With 80,000 streamlines, major memory issues arise due to browser limitations. This makes it impossible to currently load very large datasets. One could circumvent that problem by using a browser with a much higher memory limit (like the Firefox Nightly version: https://nightly.mozilla.org/). Other options are to use streamline compression (Presseau et al., 2015; Rheault et al., 2015) and grouping of similar streamlines (Garyfallidis et al., 2012) would help the framerate and fluidity of the application in the future since it equates to showing less points per streamline, thus pushing the limit of the current maximum number of streamlines.

### 3.2. Streamline interactions

Every interaction in Fiberweb works in real time. The framerate is not impacted whether or not the intersected streamlines are shown. This is because of the use of a specific shader to show only the portion intersecting the anatomy. This means that rendering the intersected streamlines is just like rendering standard streamlines.

The selection also has little impact on the framerate due to the use of an octree, and this data structure does not impact the loading time of the streamlines. However, when loading bigger streamline datasets, the framerate can drop when in areas of high streamline density during the selection. The framerate is only affected when moving the selection box, going back to normal once it is stopped.

Real-time tractography can have a bigger impact on the framerate of Fiberweb. A noticeable reduction in framerate is obtained when getting close to 1,000 seeds (10 seeds per axis). This drop is more present when tracking longer bundles. In general, the number of seeds is the limiting factor. The rest of the parameters do not negatively impact the framerate. However, using stricter parameters can speed up the algorithm since the streamlines will be shorter in general. Furthermore, just like the selection, the problems with the framerate only exist when moving the box around, since this is when the tractography is computed.

### 3.3. Probabilistic tractography : online vs. offline

The proposed probabilistic real-time tractography algorithm is based on a field of vectors (cartesian coordinates) of directions and their associated uncertainty angle. This data is extracted from fiber ODFs and is compared to probabilistic tracking based on fODF (Girard et al., 2014). In the case of the data that was used in this work, the uncertainty angles were extracted at 35% of their respective maxima value (see Figure 5).

**Figure 7:**
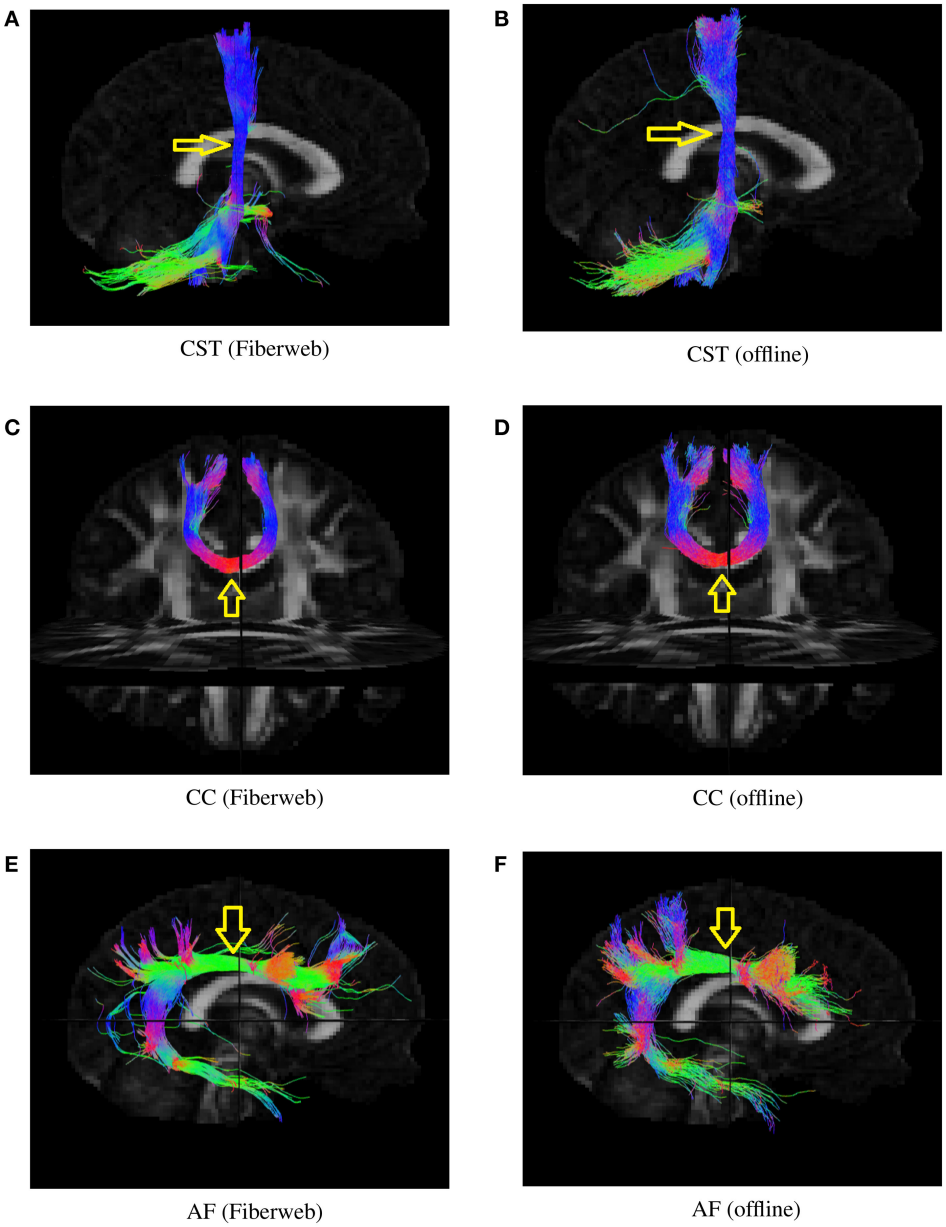
Various bundles resulting from probabilistic tractography (real-time in the Fiberweb vs offline). Dice coefficients are: CST (0.865), CC (0.928), and AF (0.811). The yellow arrows show the location of seeding regions. **(A)** Results of the Fiberweb algorithm for the corticospinal tract. **(B)** Results of the offline algorithm for the corticospinal tract. **(C)** Results of the Fiberweb algorithm for the corpus callosum. **(D)** Results of the offline algorithm for the corpus callosum. **(E)** Results of the Fiberweb algorithm for the arcuate fasciculus. **(F)** Results of the offline algorithm for the arcuate fasciculus.

To determine whether or not this proposed tracking was valid, both probabilistic algorithms were used to generate 1,000 streamlines from the same regions (Figure 7), represented by a box of 2 × 2 × 2 voxels. This was done with the help of a selection box in Fiberweb and with the use of a seeding mask for the offline algorithm. The parameters for both algorithms were the default ones. This was performed for three different bundles. The first bundle compared is the corticospinal tract (CST). It is important to note that both CSTs have projections to the cerebellum, which are most likely part of the corticopontine tract. These are not filtered out. All streamlines produced from the seed region are shown. The second compared bundle is a part of the corpus callosum (CC) under the primary motor cortex. The last bundle is the arcuate fasciculus (AF). In each case, the reconstructed bundles are qualitatively very similar. Overall, the online version always seems to produce slightly smaller and conservative bundles, with less fanning.

Bundles similarities were also quantitatively scored using a weighted Dice ratio. Table 1 shows the overlap ratio between the offline and online versions of each bundle that was evaluated. All three scores range between 81 and 93%. These are very good overlap scores, considering the fact that both tracking algorithms use a different tracking equation and that the real-time Fiberweb version only uses peaks and their uncertainty as opposed to the full fODF. This is also the reason why it is not expected to find a perfect agreement between the two algorithms. Also, this indicates that overall, the two techniques share an excellent overlap varying slightly depending on the bundle being reconstructed. This means that our real-time probabilistic tractography algorithm rapidly produces an excellent approximation to a state of the art offline probabilistic tractography.

**Table 1:** Comparison between offline and online algorithms using the Dice coefficient.

**Bundles**	**Weighted Dice coefficient**
CST	0.865
CC	0.928
AF	0.811

## 4. Discussion

Medical imaging software tools are often hard to install and hard to use for non-technical users. Fiberweb bypasses that problem by being built for the web. This makes the application cross-platform, and ready to use without the need for the installation of anything outside of having a web browser that is up-to-date. Furthermore, out of the few visualization web applications that already exist, none allow for real-time interaction and processing such as selection or real-time tractography.

### 4.1. New probabilistic algorithm

In this article, a modified version of an existing real-time tractography algorithm was introduced, making it possible to use it in a probabilistic way. This allows for a new way to visualize data in real time. It was validated qualitatively and quantitatively against a state of the art offline tractography algorithm. When compared visually, one can see that the offline version of the bundles recreated tend to look more probabilistic (more fanning and more spurious streamlines). This is due to multiple factors. It is also important to remember that both algorithms use a different tracking equation and that the real-time FiberWeb version only uses peaks and an uncertainty as opposed to the full fODF.

**Figure 8:**
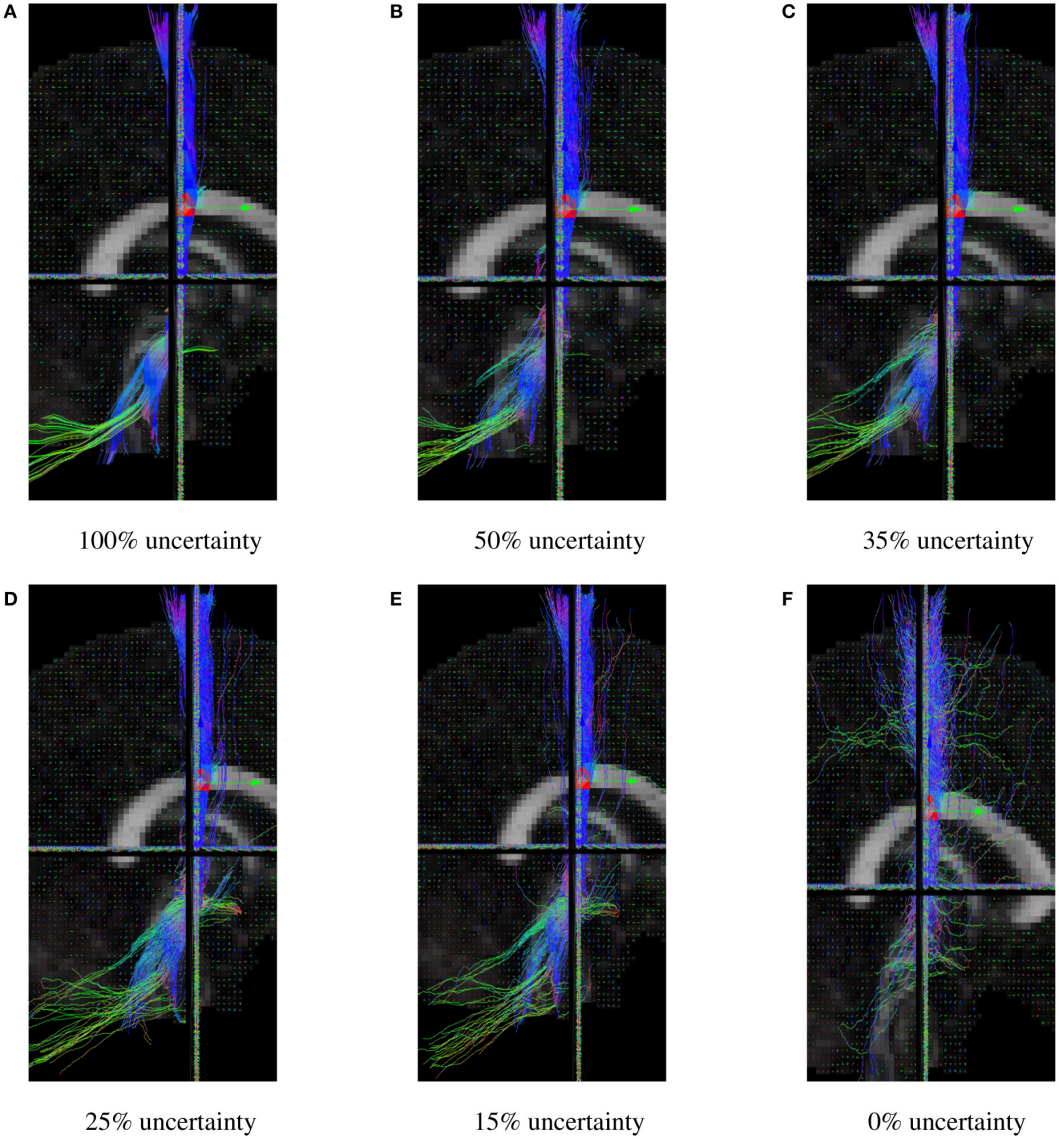
CST bundles resulting from probabilistic tractography, with a varying uncertainty percentage threshold. **(A)** Results of the Fiberweb algorithm for 100% uncertainty. **(B)** Results of the Fiberweb algorithm for 50% uncertainty. **(C)** Results of the Fiberweb algorithm for 35% uncertainty. **(D)** Results of the Fiberweb algorithm for 25% uncertainty. **(E)** Results of the Fiberweb algorithm for 15% uncertainty. **(F)** Results of the Fiberweb algorithm for 0% uncertainty.

The uncertainty angle has the biggest impact on how probabilistic the algorithm will be. Using a 100% threshold, the tractography would give results identical to the deterministic version of the algorithm. Inversely, using a 0% threshold, the tracking would take any random direction part of the fiber ODF. In our case, 35% was used because it yielded the best Dice quantitative scores, while still looking probabilistic. In Figure 8, it can be noted that lowering the uncertainty threshold induces more jaggy streamlines, while also adding more spurious streamlines.

**Figure 9:**
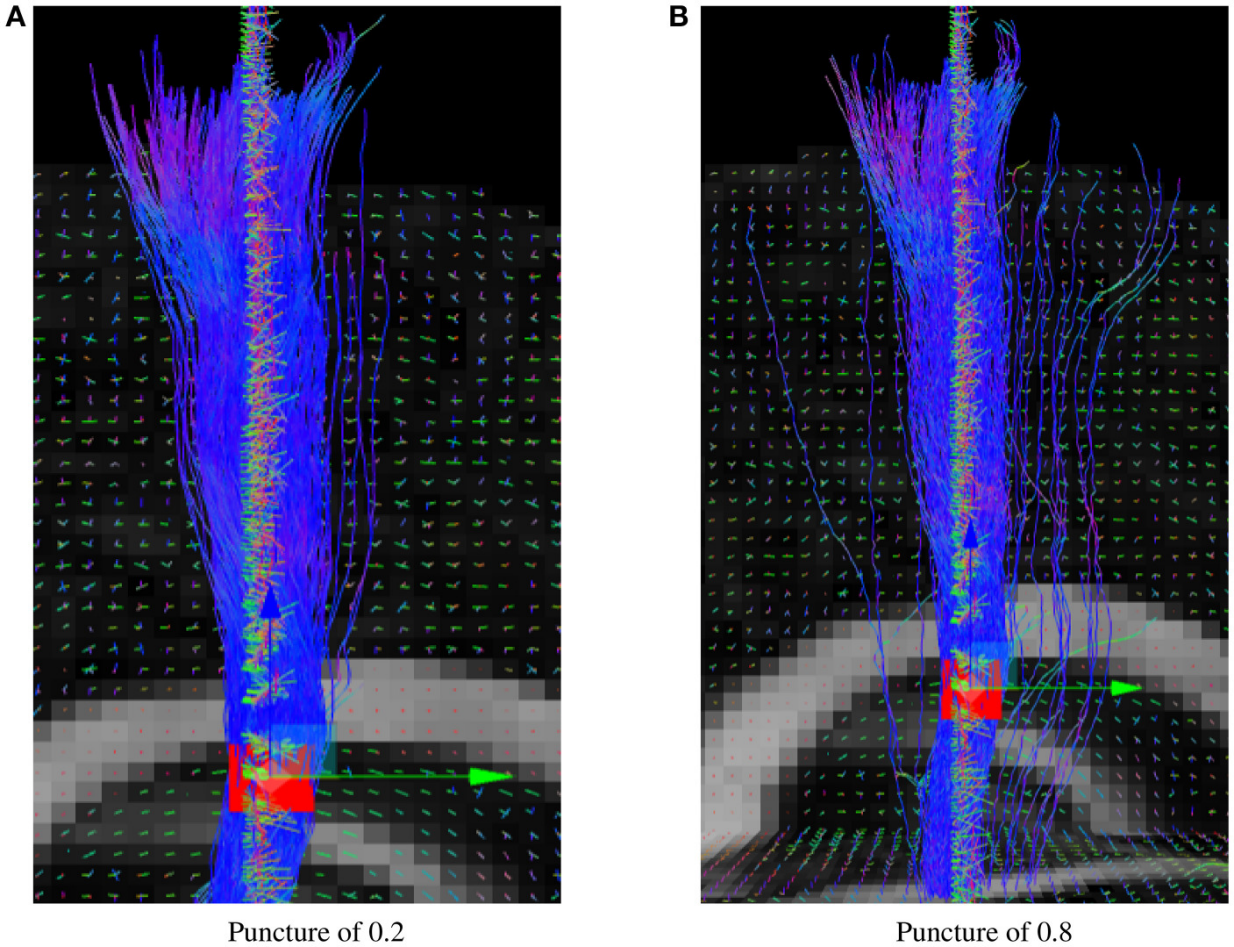
Probabilistic RTT of the CST for different puncture values. **(A)** Results of the Fiberweb algorithm with a puncture value of 0.2. **(B)** Results of the Fiberweb algorithm with a puncture value of 0.8.

The puncture parameter of the real-time tractography algorithm can modify the effect of the uncertainty angle since it differently weighs the incoming and next directions. A higher puncture value will make it look more probabilistic, but also generate more noisy spurious streamlines. Results in this paper were generated using a value of 0.2 since it is the suggested value for the deterministic real-time tractography algorithm (Chamberland et al., 2014). In Figure 9, it can be seen that the changes of direction are much smoother when the puncture value is of 0.2.

**Figure 10:**
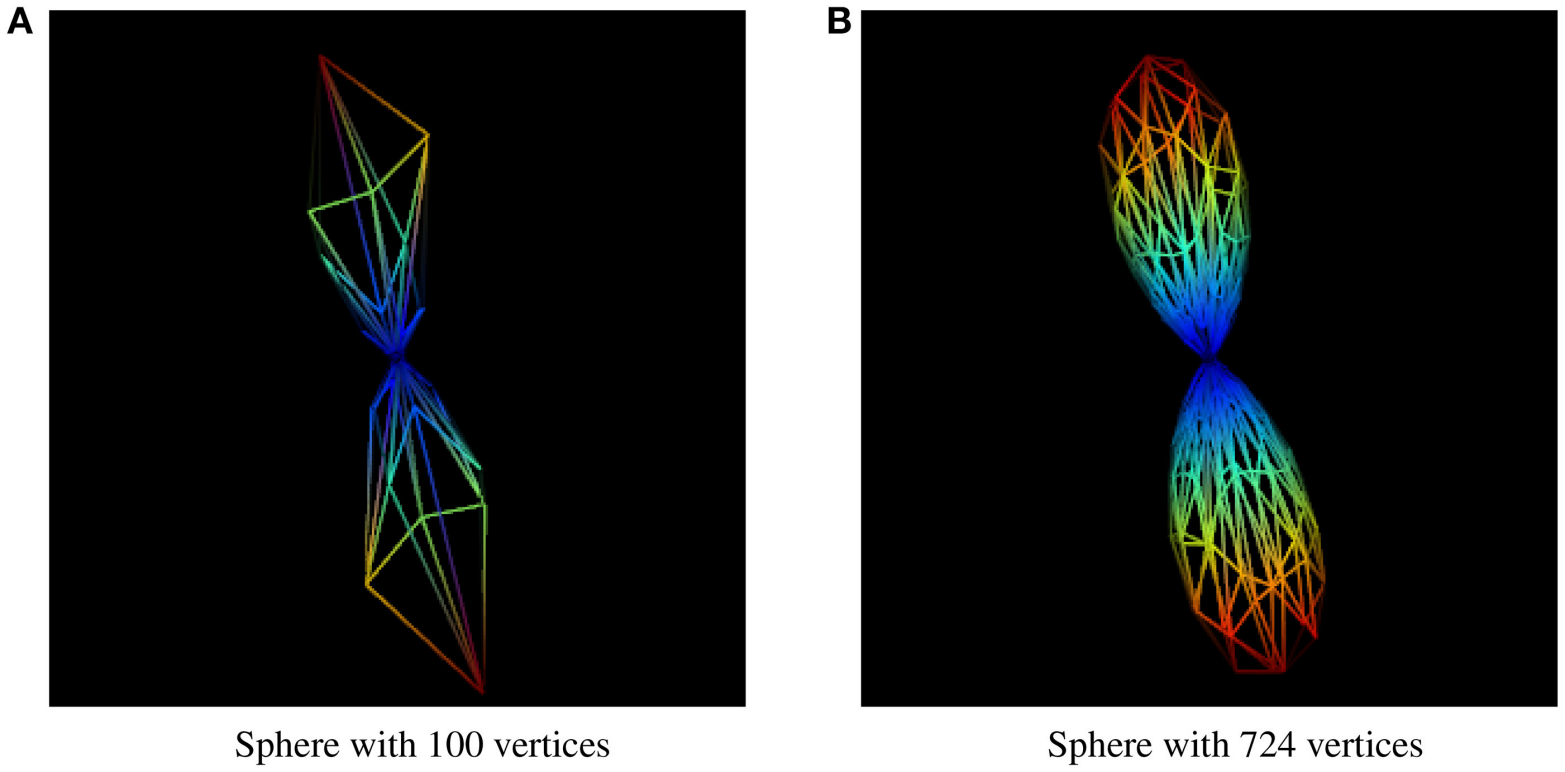
fODF represented by spheres of different resolutions. **(A)** fODF represented by a sphere of 100 vertices. **(B)** fODF represented by a sphere of 724 vertices.

The resolution of the sphere on which the fiber ODFs are mapped affects the accuracy of the uncertainty angle, even though it is the factor that has the least impact on the final result. Results presented in this paper were generated using a sphere with 724 symmetrical vertices. A sphere with a lower resolution would give us uncertainty angles that are less representative of the user-inputted percentage value of a maxima, and not even an accurate maxima. The difference between two different sphere resolutions to display the same fODF can be seen in Figure 10.

### 4.2. Applications

One of the applications of FiberWeb is quality assurance. With the possibility to access a medical visualization tool anywhere or on any platform, without the need for anything else, it will make it easier to validate and check smaller datasets. For instance, suppose one has 1,000 subjects in a tract-based white matter study. One can imagine using the FiberWeb to rapidly inspect tractograms through a web quality control interface such a CBRAIN (Sherif et al., 2014). Alternatively, before doing a full-brain tractography of a million streamlines, it would be possible to explore smaller sets of 25,000 streamlines beforehand really quickly, especially since parameters used for tractography can change depending on the subject or region (Pierpaoli et al., 1996; Chamberland et al., 2014). Furthermore, with the help of the selection feature, it would be possible to validate the presence of certain bundles.

Fiberweb could also be used as a teaching tool. For example, one could imagine a scenario where a teacher in medical school would want to illustrate a few fiber bundles to his class without having prepared for it. This could be achieved simply by opening a web page, loading his medical brain data and using the visualization, selection or real-time tracking features of Fiberweb to display the bundles. Then, by just sharing the data he used for the demonstration, every student could be able to review and explore it on their own device without the need to install an external software.

The new probabilistic algorithm allows for a new way to explore data without the need of having huge streamlines datasets, especially in the case where a specific region of interest is the sole object of the exploration. For example, in neurosurgical planning, it could be of great interest to use Fiberweb. Around regions of oedema, necrosis and mass effect due to the presence of a tumor, standard tracking parameters no longer hold. It is important to be able to reduce the FA threshold, change the step size and explore the uncertainty of the fODF peaks to present a more realistic representation of the data to the surgeon. Having both deterministic and probabilistic RTT could help more easily assess the effect of the tumor mass effect and oedema on the peaks, diffusion measures and corresponding reconstructed streamlines. It could be the first step towards showing the surgeon the most probable and improbable streamlines around and inside the tumor region of interest.

### 4.3. Future work

The first goal of Fiberweb is to support larger datasets. Unfortunately, it is mostly tied with the evolution of the varying browsers. Another main goal is making Fiberweb work just as well on tablets as in the browser. With the recent release of WebGL 2.0, one of the first task that will need to be done is to upgrade Fiberweb to use this version, mainly through the Three.js library. WebGL 2.0 will bring performance upgrades compared to the 1.0 version. For example, it will enable the use of 3D textures instead of only 2D, which is great considering anatomies are just like 3D textures. Currently, WebGL 2.0 was only announced, and it will probably be a while before it is widely available. Another major focus of Fiberweb will be to add new features. Currently, only selection is available to interact with streamlines datasets. It would be interesting to add the possibility to use more than one selection box to make the streamlines selection possibly more precise. It would also be interesting to add exclusion boxes for the same reason. Additionally, it would be great to add segment based filtering (Houde et al., 2015) to Fiberweb to make sure the selection would work well with compressed streamlines. Since performance is key, making Fiberweb multi-threaded would be a good way to gain a lot of optimization, like splitting the rendering and the algorithms into separate threads. Also, it would be interesting to add streamlines compression and clustering during load time (Rheault et al., 2015), allowing us to load bigger datasets. Finally, the last goal of Fiberweb will be to allow more file types to be used, since currently, only NIFTI and TrackVis files are supported. Additionally, while Fiberweb's user interface is usable, it still needs improvements. This will be addressed by consulting the actual users of the software to pinpoint exactly what is expected of them.

## 5. Conclusion

In this article, Fiberweb, a new browser-based medical dMRI tractography visualization tool, was presented. This web application, unlike others of its kind, allows the user to interact with his data through virtual dissection with the help of a selection box, and through streamlines reconstruction in real time. Furthermore, it introduces a new real-time probabilistic tractography algorithm. It was shown to perform very closely to a state of the art probabilistic algorithm, both qualitatively and quantitatively. This makes data exploration efficient, simple and fast. This is especially appropriate when you want to look at a smaller region in the brain, or know the effect of anomalies, like a tumor, lesion or oedema. This new tool will be particularly important to do quick quality validation imaging datasets, without the need to install any external software, which are sometimes restricted to a specific platform. Fiberweb aims to be a free to use quality assurance tool. You can see Fiberweb in action at: www.imeka.ca/fiberweb.

## Author contributions

LL has implemented both Fiberweb and the new RTT probabilistic algorithm presented by the paper. FM has participated in the development of Fiberweb. MC has helped generating the results regarding the new RTT algorithm. JH has participated in the development of Fiberweb. KW and MD supervised the project, helping design Fiberweb and its goals, as well as the new algorithm. All authors mentionned previously drafted or revised the article.

### Conflict of interest statement

The authors declare that the research was conducted in the absence of any commercial or financial relationships that could be construed as a potential conflict of interest.
