# Supplementary material for: Fiberweb: Diffusion Visualization and Processing in the Browser
Source: Front Neuroinform. 2017 Aug 18;11:54. doi: 10.3389/fninf.2017.00054 (PMC5563309; doi:10.3389/fninf.2017.00054)
Supplement: Supplementary file 1 [file Presentation1.PDF]

Supplementary Material: Fiberweb : diffusion visualization and interactivity in the browser

Louis-Philippe Ledoux*, Felix C. Morency, Martin Cousineau, Jean-Christophe Houde, Maxime Descoteaux* and Kevin Whittingstall

*Correspondence:
Louis-Philippe Ledoux:
louis-philippe.ledoux@usherbrooke.ca

Maxime Descoteaux: m.descoteaux@usherbrooke.ca

1 SUPPLEMENTARY CODE

Here is the code allowing us to compute peaks of the fODFs, as well as their uncertainty alpha parameter.

```
#! /usr/bin/env python
from __future__ import print_function
import os
import numpy as np
import nibabel as nib
import math

import argparse
import os
import logging
import warnings

import time
from dipy.reconst.shm import sph_harm_lookup
from dipy.core.sphere import Sphere
from dipy.data import get_sphere
from dipy.reconst.peaks import peak_directions
from dipy.core.ndindex import ndindex

def load(input) :
    img = nib.load(input)
    return img.get_data(), img.affine

def find_order(data) :
    return int((-3 + np.sqrt(1 + 8 * data.shape[-1])) / 2)


def get_B_matrix(order, sphere, basis_type) :
    sph_harm_basis = sph_harm_lookup.get(basis_type)
    if sph_harm_basis is None:
```

```
        raise ValueError("Invalid basis name.")
    B, m, n = sph_harm_basis(order, sphere.theta, sphere.phi)
    return B


def get_maximas(data, sphere, B, threshold) :
    sf = np.dot(data, B.T)
    if False:#sf.sum() != 0:
        print(sf)
    directions, values, indices = peak_directions(sf, sphere, threshold)
    if False:#sf.sum() != 0:
        print(values)
        itemIndex = np.where(sf == values)
        print(itemIndex)
        print(indices)
        input()
    return directions, values, indices

def get_uncertainty(data, sphere, B, maximas, uncertaintyThreshold) :
    odf = np.dot(data, B.T)
    angles = np.array(())
    for aMaxima in maximas :
        #Init arrays
        verticesProcessed = np.array(())
        verticesToProcess = np.array(())
        verticesToProcess = np.insert(verticesToProcess,0,aMaxima)
        #Init scalars
        maximaOdfValue = odf[aMaxima]
        threshold_not_met = True
        bestOdfValue = maximaOdfValue
        bestVertex = aMaxima
        bestDifferenceValue = maximaOdfValue
        targetValue = (uncertaintyThreshold * maximaOdfValue)

        while threshold_not_met and verticesToProcess.shape[0] > 0 : ##Full Width Half Max
            #Init array
            neighboursToAdd = np.array(())

            #Check for best result
            for aVertex in verticesToProcess :
                if abs(odf[aVertex] - targetValue) < bestDifferenceValue :
                    bestOdfValue = odf[aVertex]
                    bestVertex = aVertex
                    bestDifferenceValue = abs(odf[aVertex] - targetValue)

                #Find neighbours
                if odf[aVertex] > (targetValue - 0.000001) :
                    neighboursToAdd = get_neighbour_vertices(aVertex, sphere,
                      verticesProcessed, neighboursToAdd, verticesToProcess)
```

```
            #Handle vertices
            verticesProcessed = np.concatenate((verticesProcessed,verticesToProcess),axis=0)
            verticesToProcess = neighboursToAdd

        if aMaxima == bestVertex :
            angles = np.insert(angles,angles.shape[0],0)
        elif uncertaintyThreshold < 0.000001 :
            angles = np.insert(angles,angles.shape[0],math.pi/2)
        else :
            angles = np.insert(angles,angles.shape[0],get_angle( np.array([0,0,0]),
                sphere.vertices[aMaxima], sphere.vertices[bestVertex] ))

    return angles

def get_angle( sphereCenter, peakPos, halfMax ) :
    v1 = np.subtract(peakPos, sphereCenter)
    v2 = np.subtract(halfMax, sphereCenter)
    return math.acos(np.dot(v1,v2) / (np.linalg.norm(v1) * np.linalg.norm(v2)))

def get_neighbour_vertices(aVertex, aSphere, aVerticesProcessed,
   aNeighboursIndex, aVerticesToProcess) :
    neighboursIndex = aNeighboursIndex
    listOfFaces = np.where(aSphere.faces == aVertex)
    for i in range(listOfFaces[0].shape[0]) :
        faceId = listOfFaces[0][i]
        aVertexId = listOfFaces[1][i]
        for j in range(3):
            currentIndex = aSphere.faces[faceId][j]
            if j != aVertexId and
               np.where(neighboursIndex == currentIndex)[0].shape[0] == 0 and
               np.where(aVerticesProcessed == currentIndex)[0].shape[0] == 0 and
               np.where(aVerticesToProcess == currentIndex)[0].shape[0] == 0 :
                neighboursIndex = np.insert(neighboursIndex,neighboursIndex.shape[0],
                 currentIndex)

    return neighboursIndex

def get_maps(data, mask, args) :
    maxNbOfMaximas = 20
    nufo_map = np.zeros(data.shape[0:3])
    afd_map = np.zeros(data.shape[0:3])
    uncertainty_map = np.zeros((data.shape[0],data.shape[1],data.shape[2],maxNbOfMaximas))
    order = find_order(data)
    sphere = get_sphere(args.number)
    B = get_B_matrix(order, sphere, args.basis)

    count = 0
```

```
    for index in ndindex(data.shape[0:3]) :
        count += 1
        if mask[index] > 0 :
            if np.isnan(data[index]).any() :
                print('NaN')
            else :
                maximas, afd, indices = get_maximas(data[index], sphere, B, args.threshold)
                angles = get_uncertainty(data[index], sphere, B, indices, args.uncertainty)
                angles = np.expand_dims(angles,axis=1)
                awesomeIndices = np.argsort(angles,axis=0)
                awesomeIndices = awesomeIndices[::-1]
                angles = angles[awesomeIndices[:,0]]
                maximas = maximas[awesomeIndices[:,0]]

                directionsPlusAngles = np.concatenate((maximas,angles),axis=1).flatten()
                if directionsPlusAngles.shape[0] < maxNbOfMaximas:
                    directionsPlusAngles = np.concatenate((directionsPlusAngles,
                        np.zeros(maxNbOfMaximas-directionsPlusAngles.shape[0])),axis=0)
                if directionsPlusAngles.shape[0] > maxNbOfMaximas:
                    directionsPlusAngles = directionsPlusAngles[0:maxNbOfMaximas]

                uncertainty_map[index] = directionsPlusAngles
        print('Progress [' + str(int(count / float(data.size/data.shape[3]) *
                      100)) + '%]...', end='\r')

    return  uncertainty_map


DESCRIPTION = """
    Script to compute the uncertainty peaks map from fiber ODFs.
"""

def buildArgsParser():

    p = argparse.ArgumentParser(description=DESCRIPTION)

    p.add_argument('input', action='store', metavar='fODFs', type=str,
                   help='Path of the fODF volume in spherical harmonics (SH).')

    p.add_argument('--basis', action='store', dest='basis',
                   metavar='string', required=False, default='fibernav',
                   type=str, help='Basis used for the SH coefficients. Must \
                   be either mrtrix or fibernav (default).')

    p.add_argument('--mask', action='store', dest='mask',
                   metavar='', required=False, default=None, type=str,
                   help='Path to a binary mask. Only the data inside the mask \
                   will be used for computations and reconstruction.')
```

```
    p.add_argument('-t', dest='threshold', action='store', type=float, default='0.25',
                   help='Threshold on fodf amplitude in percentage (default : 0.25)')

    p.add_argument('-n', dest='number', action='store', type=str, default='symmetric724',
                   help='Number of directions
                     uniformly distributed on sphere (default : 100)')

    p.add_argument('-u', dest='uncertainty', action='store', type=float, default=1.0,
                   help='Uncertainty percentage /1.0')

    p.add_argument('-f', action='store_true', dest='overwrite', required=False,
                   help='If True, the saved files volume will be overwritten \
                   if they already exist.')

    return p


def main():
    parser = buildArgsParser()
    args = parser.parse_args()

    tps1 = time.clock()

    # Load input image
    data, affine = load(args.input)

    if args.mask is None :
        mask = np.ones(data.shape[0:3])
    else :
        mask, affine2 = load(args.mask)

    # Compute uncertainty map
    uncertainty_map = get_maps(data, mask, args)

    # Save result
    img = nib.Nifti1Image(np.array(uncertainty_map, 'float32'),  affine)
    nib.save(img, 'maximasUncertainties'+ str(int(args.uncertainty*100)) + '.nii')


    tps2 = time.clock()
    print('Time elapsed : ' + str((tps2 - tps1)/60.0) + ' min.')


if __name__ == "__main__":
    main()
```

Here is the pseudo-code representing the algorithm allowing us to do the probabilistic real-time tracking based on the peaks map.

```
PerformRTT()
{
    for each seeds
    {
        // This seed is our starting point
        var currPosition = currentSeed;

        // We need to track forward and backward to form a single fiber
        var aFiberF = RttAlgorithm(currPosition, 1.0 );
        aFiberF.reverse();

        var aFiberB = RttAlgorithm(currPosition, -1.0 );

        var aFiber = aFiberF.concat(aFiberB);
        // If the fiber is valid, we add it to the array of fibers to display
        if(isFiberValid())
        {
            aFibersArray.push(aFiber);
        }
    }

    return aFibersArray;
};

RttAlgorithm(initialPosition, mainDirection)
{
    var currentPosition = initialPosition;
    var currentFa = GetMaskValueFromPosition(currentPosition);

    if (IsPositionInMask(currentPosition) && currentFa > faThreshold)
    {
        var currentDirection = PickDirection(currentPosition);
        Normalize(currentDirection);
        currentDirection *= mainDirection;

        while (true)
        {
            var nextPosition = currentPosition + stepSize * currentDirection;

            currentFa = GetMaskValueFromPosition(nextPosition);

            if (!IsPositionInFAMask(nextPosition) || currentFa < faThreshold)
            {
                break;
            }

            var nextDirection = AdvecIntegrate(currentDirection, nextPosition,
                currentFa, puncture);
```

```
            if (DotProduct(currentDirection, nextDirection) < 0.0)
            {
                // Ensures the two vectors have the same directions
                Flip(nextDirection);
            }

            var angle = GetAngle(currentDirection, nextDirection);
            if (angle > angleThreshold)
            {
                break;
            }

            points.push(currentPosition);


            currentPosition = nextPosition;
            currentDirection = nextDirection;
        }
    }
    return points;
};

AdvecIntegrate(currentDirection, currPosition, fa, puncture)
{
    var peaks = GetPeaksFromPosition(currPosition);
    var angleMin = 360.0;
    var out;

    // Find the best next direction
    for each peaks
    {
        Normalize(peak);
        if (DotProduct(currentDirection, peak) < 0)
        {
            Flip(peak);
        }

        var angle = GetAngle(peak, currentDirection);
        if (angle < angleMin)
        {
            angleMin = angle;
            out = peak;
        }
    }

    // Affect next direction by its uncertainty angle
    var uncertaintyAngle = Math.random() * peak.alpha;
    var zAngle = Math.random() * 360.0;
```

```
    ApplyUncertaintyRotation(out,uncertaintyAngle,zAngle);

    var result =  out * fa +
       (1.0 - fa) * (currentDirection * (1.0 - puncture ) + out * puncture);
    Normalize(result);

    return result;
};
```

2 WEIGHTED DICE COEFFICIENT

In order to measure the overlap of two bundles reconstructed differently, we used a slightly modified version of the Dice coefficient (Dice (1945)), as proposed by Cousineau et al. (2016). Let W_i be a bundle reconstructed using a certain tracking algorithm and W_j another bundle extracted from another algorithm. Note that in this study, W_i and W_j are the same anatomical bundle. The standard Dice coefficient between W_i and W_j is

$$D(W_i, W_j) = \frac{2\sum_v (W_i \cap W_j)_v}{\sum_v W_{i,v} + \sum_v W_{i,v}}. \tag{S1}$$

where W_i and W_j contain binary values (1 inside the bundle and 0 otherwise) and v is a voxel index. As is, the Dice coefficient greatly penalizes for spurious streamlines that would be far from the core of the bundle. Given that white matter bundles have more tracts in the middle than in their periphery, Cousineau et al. proposed a weighted Dice coefficient which accounts for the number of streamlines per voxel. In that perspective, each voxel in W_i and W_j contains a value between 0 and 1 expressing the fraction of tracts passing through that position. The weighted Dice metric sums the voxels that overlaps in W_i and W_j and divide by the total sum of voxels:

$$D(W_i, W_j) = \frac{\sum_{v'} W_{i,v'} + \sum_{v'} W_{i,v'}}{\sum_v W_{i,v} + \sum_v W_{i,v}}, \tag{S2}$$

where v' stands for the voxels that are within the intersection of the W_i and W_j bundles. The weighted Dice gives a larger importance to areas with dense fibers.
